# Testing Passenger Car Brake Pad Exploitation Time’s Impact on the Values of the Coefficient of Friction and Abrasive Wear Rate Using a Pin-on-Disc Method

**DOI:** 10.3390/ma15061991

**Published:** 2022-03-08

**Authors:** Andrzej Borawski

**Affiliations:** Faculty of Mechanical Engineering, Bialystok University of Technology, 45C Wiejska Str., 15-351 Bialystok, Poland; a.borawski@pb.edu.pl

**Keywords:** mechanical engineering, brake pads, friction, wear, tribological properties, pin-on-disc

## Abstract

The braking system is one of the most important components in any motor vehicle. Its proper function in emergency situations may save road users’ lives. Today, as vehicles have more and more power at their disposal, leading to increased acceleration and maximum speed, the issue of effective braking is particularly important. It must also be noted that brakes are used in harsh conditions (water and salt, especially during winter), and must provide appropriate durability (on average, circa 30,000 km). For these reasons, many institutions conduct research aimed, among other things, at minimizing fading. However, this study looked into a different matter, focusing on how the operating conditions mentioned above, including the lifespan of brakes, impact the tribological properties of the friction pair. To achieve this, samples from brake pads were obtained (both brand new and used). Next, using a pin-on-disc tribological test, it was shown that the pads have lower coefficients of friction and abrasive wear rates. The results indicated that both parameters change in a manner that is dependent on how long the brake system has been in use.

## 1. Introduction

Brakes constitute one of the most important components in a vehicle. The life and health of the driver, passengers and other road users may depend on the proper and effective functioning of breaks. For these reasons, extensive research is conducted in order to identify the problems concerning the construction and action of brakes, and to improve them. The most common brake system is the friction disc brake, which replaced the older drum brake design [1,2,3,4]. Brakes use friction to transform mechanical energy into thermal energy [5,6,7]. Heat is then transferred to the atmosphere and other components of the brake system and suspension of the vehicle [8].

The efficiency of a brake is mainly determined by the coefficient of friction between the pads and the brake disc. Unfortunately, the coefficient is not a constant value—this is a known problem in various research streams [9,10,11,12,13]. Significant changes in temperature of the brake system’s working parts may cause the coefficient of friction between the disc and pad to fall, in some cases, almost to zero [14]. In addition, the humidity in the close surroundings of the friction pair is important. Increased humidity may cause condensation and create a water film. In such cases, at low operation speed, the modulation of the value of the coefficient of friction may reach up to 30% [15].

**Figure 1:**
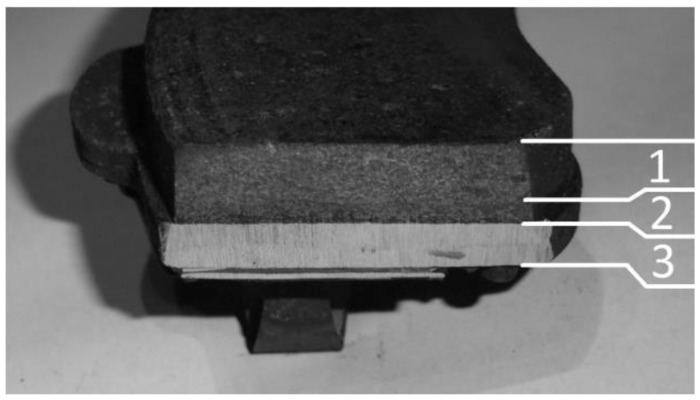
Cross-section of the real-life object of study: **1**—friction material; **2**—adhesive layer (interlayer); **3**—back plate.

However, the biggest impact on the friction force is made by the composition of the friction pair. Brake discs are, in most cases, made from grey cast iron, as it is characterized by good thermal conductivity and anti-vibration capacity [16,17]. Matters are different for brake pads. Brake pad manufacturers are using approximately 2000 different materials [18], which have different effects of the final product. An average brake pad is made from 10 to 20 different substances. In addition, the proportions of these materials are not homogeneous across the entire section of the pad. Brake pads show a clear division (resulting, among other things, from production technology) into the friction layer, the interlayer (adhesive) and the back plate (Figure 1) [19]. This structure causes the composition of the friction material to change as the brake pad runs out, which has a direct impact on the process of braking [20,21].

Harsh operating conditions, resulting from frequent and significant changes of temperature, as well as the corrosive environment in which the brake system works (water and salt, especially during winter) may, in time, result in a permanent change of the friction material’s structure, leading to changes in the tribological properties of the brake pads and discs. Consequently, the braking force may fall, increasing the distance necessary to stop the vehicle [4,22,23,24,25].

The main purpose of this study was to find out if and how the tribological properties (such as the coefficient of friction and the abrasive wear rate) of brake pads change with exploitation time.

## 2. Materials and Methods

**Figure 2:**
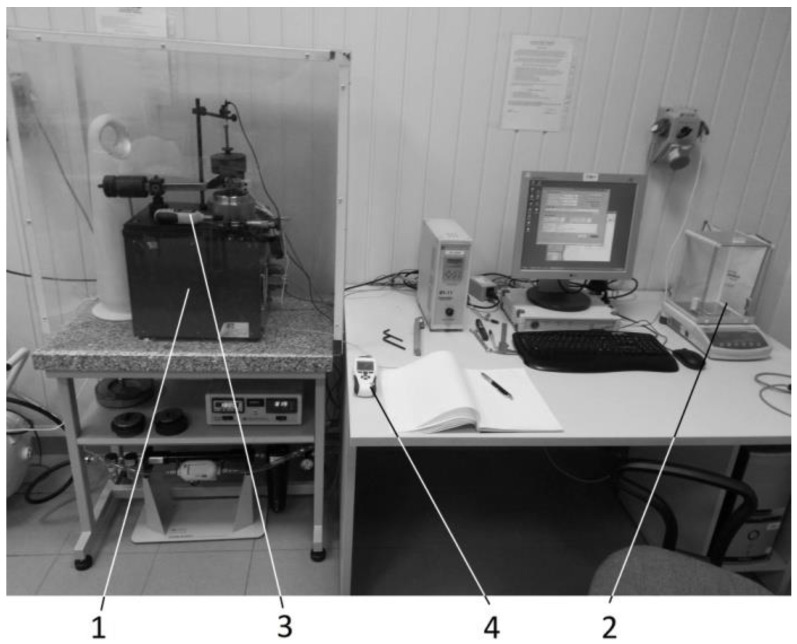
Test station: **1**—T-11 tribological tester; **2**—RADWAG WAS 160/C/2O scale; **3**—MT886 hygrometer; **4**—Velleman DEM106 temperature sensor.

The research was conducted using a T-11 tribological pin-on-disc tester (Figure 2). This method may be applied in dry friction tests as well as with the use of lubricants [26,27]. With the use of an environmental chamber, the experiments could be conducted in the presence of various gasses or humidity values [28]. The station made it possible to determine the average coefficient of friction between a friction pair, and to evaluate the wear rate of the friction surfaces. The first parameter was measured directly during the experiment as a function of time or number of disc rotations, while the second parameter was determined on the basis of the weight change (loss) of the sample. The necessary data were obtained by weighing the sample before and after the test. The samples were weighed using a RADWAG WAS 160/C/2O scale, with a measurement accuracy of 0.1 mg. Of course, the sample had to be carefully cleaned before weighing; this is usually conducted using products such as acetone or washing benzene, and failure to take this step can lead to the results being seriously flawed [29,30,31,32].

The parameters for the experiment, i.e., the velocity, the friction path, the touch diameter, and the load on the friction pair, were constant for all samples and had the following values: velocity *v* = 1 m/s, path *S* = 1000 m, touch diameter *d* = 18 mm, and load *m* = 5 kg. The tests were carried out at an air humidity of 35% and an ambient temperature of 21 °C. These parameters were measured using a MT886 hygrometer and a type K thermocouple connected to a Velleman DEM106 sensor.

**Figure 3:**
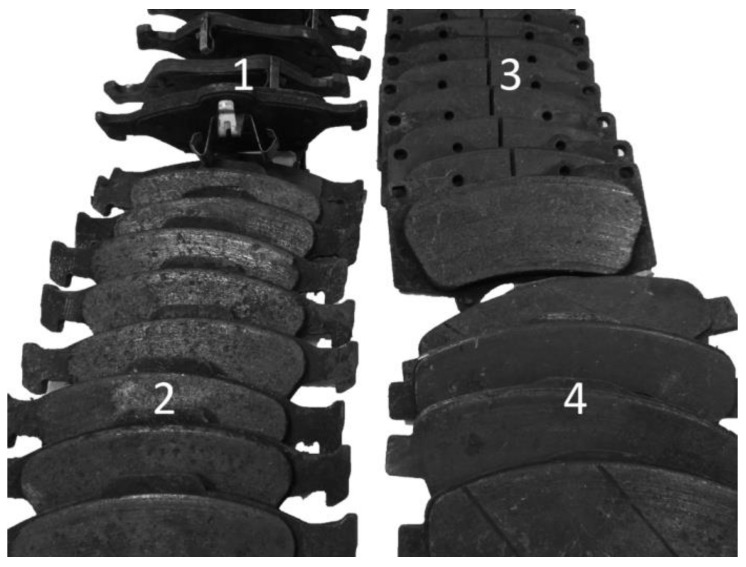
Examples of brake pads used for sampling: **1**—brake pads for small urban hatchbacks; **2**—brake pads for premium cars; **3**—brake pads for off-road vehicles; **4**—brake pads for delivery vans.

The study was conducted on samples collected from genuine brake pads, both brand new and at certain levels of wear (Figure 3 presents some of the pads used for sampling). Four groups of brake pads were used, intended for different car classes: small urban hatchbacks (group 1), premium cars (group 2), off-road vehicles (group 3) and delivery vans (group 4). The brake pads were obtained courtesy of Authorised Auto Service.

Geometrically, the samples were 1” (25.4 mm) cylinders. Three samples were cut from each pad. This produced a total of 111 samples, as detailed in Table 1. The wear of the brake pad was determined by measuring the thickness of the friction material and comparing it against the thickness of the friction surface in a brand-new pad; 0% wear describes a brand-new pad, while 100% wear means a pad with no friction material whatsoever (completely worn). As can be seen from the Table 1, in some groups, it was impossible to obtain pads that were nearly new. This is because it is uncommon to replace brake pads after such a short period of use.

The chemical composition of individual groups of pads and their layers was determined using an electron microscope equipped with an EDS detector (SDD type). The collected data are presented in Table 2.

Next, using an WPM Leipzig HPO 250 hardness tester, the hardness of individual groups of samples was determined. Each sample group was measured five times. The arithmetic mean was rounded to the full value. The results are shown in the Table 3. The obtained values were quite large; however, as shown in the literature, the hardness of friction materials may be similar or even much higher [33,34]. The differences between the hardness in different layers of the pad were also noticed by other researchers [35].

**Figure 4:**
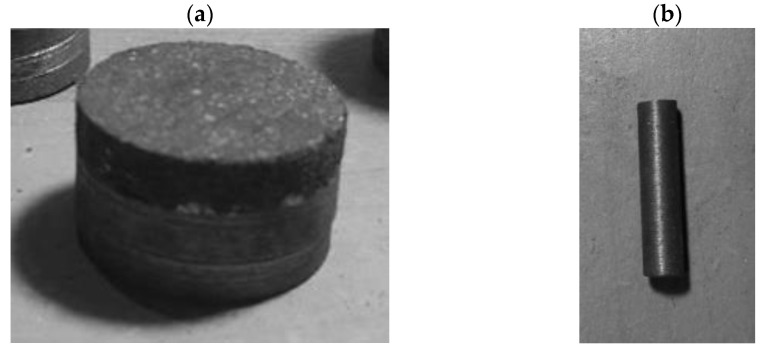
Picture of (**a**) one of the samples (disc) cut from a brake pad; (**b**) sample pin.

The counter sample, or the pin, was a 6 mm cylinder made from grey cast iron (Zl250), which is the material frequently used in brake discs. As wear of the brake pad determined the thickness of the sample, it was necessary to use different thicknesses of the pin, in order to maintain proper contact geometry. Examples of samples and pins are presented in Figure 4.

To ensure the correct contact of the sample and the counter-sample, the contact surface of the pin was polished before each test with 1200 grit sandpaper attached to the disc. Grinding consisted in activating the station for about 5 min. After this time, the sandpaper was disassembled and the proper test was started.

## 3. Results and Discussion

**Figure 5:**
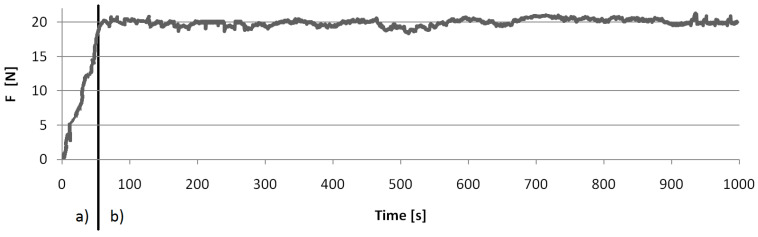
Example time profile of the friction force obtained during one of the tests: (**a**) running-in; (**b**) measurement period proper.

The direct results of the conducted tests were the values of friction force between the sample and counter sample. The software of the test station automatically recorded the values every 0.5 s, giving 2000 points of measurement and making it possible to determine the friction force time profiles (Figure 5). This made it possible to distinguish the initial runin and proper friction.

For each test, the arithmetical mean was calculated from the force *F* measured during proper friction (ignoring the run-in time). As three samples were collected from each pad, the final result is the average value from each measurement. Next, applying the Amontons–Coulomb friction law [36], the coefficient of friction was calculated, which in this case equaled:

(1) $$\mu_{i}=\frac{{\bar{F}}_{i}}{m\cdot g}$$

where: *µ*—coefficient of friction of sample *i* (where *i* = *I* … *IV*); *F*—measured average friction force of sample *i*; *g*—gravitational acceleration; *m*—pin loading mass. The resulting coefficient of friction calculations are presented in Table 4.

The single-factor analysis of variance method [37,38] was used to analyze the test results. It compares the variability between the groups to the variability within the groups. It was assumed that the confidence level would be α = 95%. The degrees of freedom of individual groups of measurements were calculated from the following relationships:

- For the qualitative factor:
  (2) $$D_{fa}=a-1$$
- For random error:
  (3) $$D_{fe}=N-a$$
- For total variation:
  (4) $$D_{ft}=N-1$$

where: *a*—the number of objects in the entire experiment; *N*—the number of experimental units in the entire experiment. If the calculations were correct, the relationship below would necessarily be true:

(5) $$D_{fa}+D_{fe}=D_{ft}$$

The next step was to calculate the sum of squares based on the results of the experiment, using the formulas below:

- For the qualitative factor:
  (6) $$SS_{a}=\overset{a}{\underset{i=1}{\sum }}n\left({\bar{x}}_{i}-\bar{x}\right)$$
- For random error:
  (7) $$SS_{e}=\overset{a}{\underset{i=1}{\sum }}\overset{n_{i}}{\underset{j=1}{\sum }}\left(x_{ij}-{\bar{x}}_{i}\right)$$
- For total variation:
  (8) $$SS_{t}=\overset{a}{\underset{i=1}{\sum }}\overset{n_{i}}{\underset{j=1}{\sum }}\left(x_{ij}-\bar{x}\right)$$

where: *n*—number of repetitions; ${\bar{x}}_{i}$—object mean; $\bar{x}$—overall mean; *x*—value of a single measurement for sample no. In addition, there needed to be a relation between *SS* values:

(9) $$SS_{a}+SS_{e}=SS_{t}$$

Mean squares calculations took the form:

- For the qualitative factor:
  (10) $$MS_{a}=SS_{a}/D_{fa}$$
- For random error:
  (11) $$MS_{e}=SS_{e}/D_{fe}$$

The above calculations made it possible to determine Fisher function values for each series of tests:

(12) $$F_{f}=\frac{MS_{a}}{MS_{e}}$$

From the statistical tables, taking into account the values calculated above and the *α* degree of confidence, the critical values for individual groups of samples were read:

(13) $$F_{crit}=F_{\propto ,D_{fa},D_{fe}}$$

The results of the above calculations are presented in Table 5.

A statistically significant influence of the operating time on the value of the coefficient of friction between the working elements of the braking system was found. This was confirmed by both the empirical *F_f_* values calculated for each group of samples, satisfying in each case the relationship:

(14) $$F_{I\ldots IV}>F_{crit}$$

as well as *p*-values. Therefore, for the confidence level *α*, the zero hypothesis read as follows:

(15) $$H_{0}:\mu_{1}=\mu_{2}=\ldots =\mu_{33}$$

where: *μ*_(1…33)_—test result for each group of samples (*I* … *IV*), has been rejected. Therefore, in order to check the degree of homogeneity of variation in the groups, the Levene test [39] was used. The final test result (Table 6) describes the relationship:

(16) $$F_{Lev}=\frac{\sum_{i=1}^{a}n_{i}{\left({\bar{x}}_{i}-\bar{x}\right)}^{2}/\left(a-1\right)}{\sum_{i=1}^{a}\sum_{j=1}^{n_{i}}n_{i}{\left({\bar{x}}_{ij}-\bar{x}\;\right)}^{2}/\sum_{i=1}^{a}\left(n_{i}-1\right)}$$

This made it possible to obtain the following results:

For the level of significance:

(17) p=100−α

it turned out that the *F_Lev_* value for the second group of samples slightly exceeded the critical value. In such cases, the heterogeneity of the variation of laboratory test results may indicate the influence of another, unknown factor on the measurement values. This factor may have been, for example, the method or conditions of operation of the vehicle from which the brake pads were obtained. In the remaining groups of samples (especially in group III), the high degree of homogeneity made it possible to state that the only factor influencing the value of the friction coefficient was the degree of wear. A more detailed analysis is planned in future studies for which preparations have already been started.

**Figure 6:**
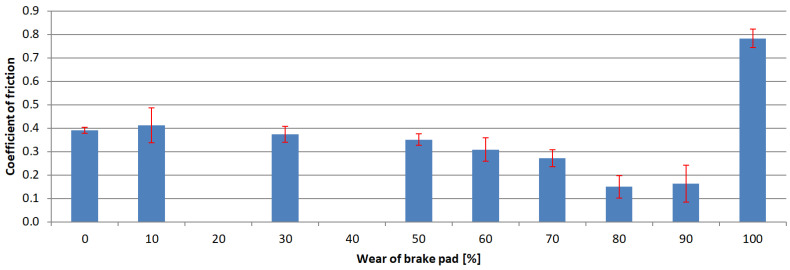
Coefficient of friction values of samples obtained from 1st brake pad group.

**Figure 7:**
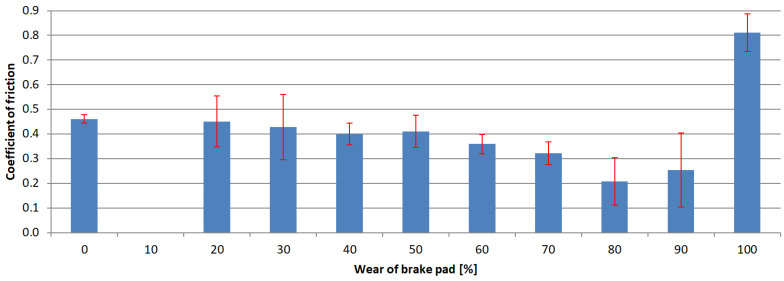
Coefficient of friction values of samples obtained from 2nd brake pad group.

**Figure 8:**
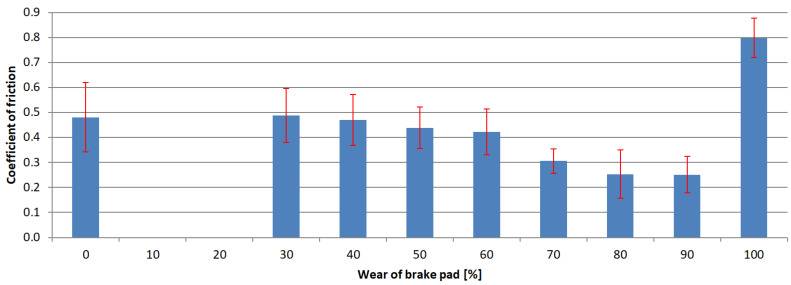
Coefficient of friction values of samples obtained from 3rd brake pad group.

**Figure 9:**
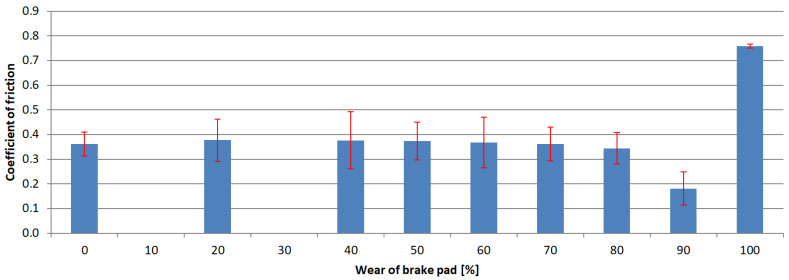
Coefficient of friction values of samples obtained from 4th brake pad group.

The next step of the analysis was to calculate the arithmetic mean value for each test series and the standard deviations. The results are presented in Figure 6, Figure 7, Figure 8 and Figure 9. Vertical bars represent the 0.95 confidence interval.

The range of point errors adjacent to the results that were missing due to the unavailability of samples allowed for an approximate interpolation. The Lagrange algorithm [40] was used for this purpose:

(18) $$W_{n}\left(x\right)=\overset{m}{\underset{k=0}{\sum }}L_{i}\left(x\right)f\left(x_{i}\right)$$

where:

(19) $$L_{i}\left(x\right)=\overset{n}{\underset{\begin{array}{c} l=0\\ k\neq l \end{array}}{\prod }}\frac{x-x_{l}}{x_{k}-x_{l}}$$

**Figure 10:**
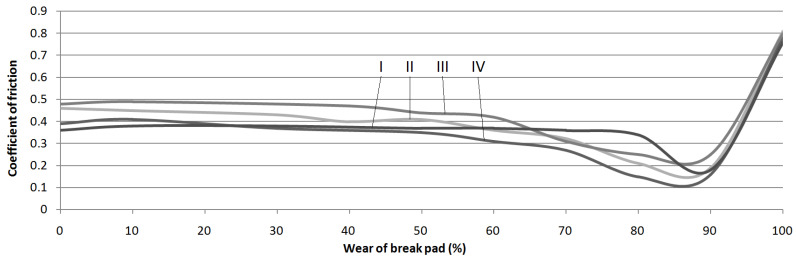
Results of calculations of the coefficient of friction of samples obtained from brake pads at various degrees of wear.

After transformation, the following was obtained:

(20) $$W_{n}\left(x\right)=\overset{m}{\underset{k=0}{\sum }}f\left(x_{i}\right)\frac{\omega_{n}\left(x\right)}{\left(x-x_{l}\right){\frac{\omega_{n}\left(x\right)}{x-x_{l}}|}_{x=x_{l}}}=\overset{m}{\underset{k=0}{\sum }}f\left(x_{i}\right)\frac{\omega_{n}\left(x\right)}{\left(x-x_{l}\right)\omega_{n}^{’}\left(x_{l}\right)}$$

where:

(21) $$\omega_{n}\left(x\right)=\left(x-x_{0}\right)\left(x-x_{1}\right)\ldots \left(x-x_{n}\right)$$

and $\omega_{n}^{’}\left(x_{l}\right)$—derivative of the polynomial $\omega_{n}\left(x\right)$ with the point $x_{l}$ being its zero point. Using the available empirical data, the presumed values of the missing measurement points were established (illustrated by the juxtaposition in Figure 10).

The analysis of the results indicated that the exploitation time affected the coefficient of friction in every case. The highest decrease in the coefficient was observed in samples where the friction material was completely worn, and the friction pair was composed of the interlayer and the pin. In these cases, the samples collected from hatchback brake pads showed a coefficient of friction at the 0.15 level.

Similar gradients of the coefficient of friction were obtained by Hagesh et al. [41]. They gave the percentage change in the content of solid lubricants and fibrous materials as the reason for the non-constant value of COF. A similar statement was provided by Coronado [42]. However, in the studied case, the composition of the individual layers was rather unchanged. It changed only with the layer. Therefore, the first reason for a drop in the COF values is a change of individual layers’ chemical compositions.

**Figure 11:**
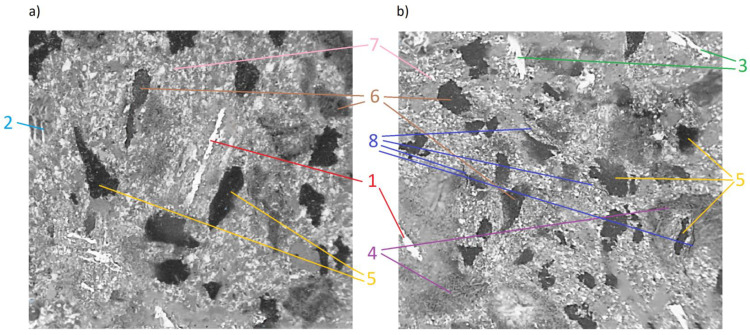
SEM micrograph of samples from same group: (**a**) 10% wear; (**b**) 80% wear. **1**—steel fiber; **2**—glass fiber; **3**—cast iron fiber; **4**—zeolites; **5**—graphite; **6**—rubber; **7**—barite; **8**—microcracks.

In order to determine whether there was any other cause of such a large decrease in the COF values, microscopic examinations were performed with the SEM technology. The photograph (Figure 11) clearly shows microcracks in the sample’s composite structure. The likely cause of these cracks was the rapid and repeated heating and cooling of the friction material. The thermal expansion coefficients of individual materials and, thus, all components, differ significantly [43,44]. Amounts of thermal energy generated during braking cause cyclic stresses and deformations [45], especially in the presence of the friction force [42]. Therefore, it was assumed that they were responsible for the microcracks.

**Figure 12:**
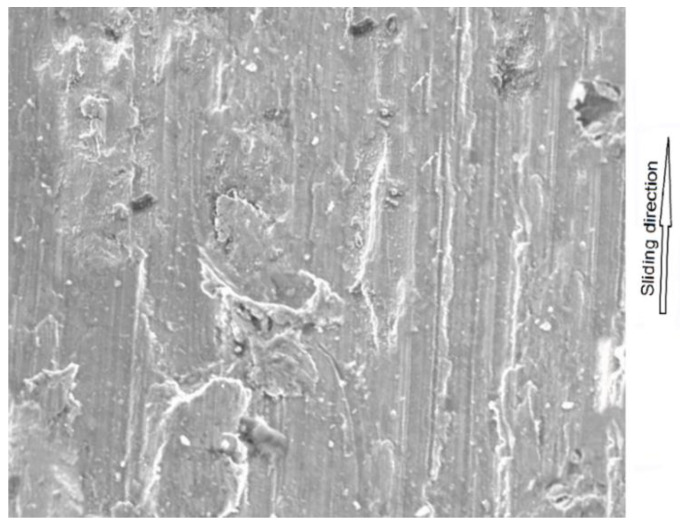
SEM micrograph of one of the back plate sample friction path.

In each of the analyzed samples, there was a significant increase in the parameter at the final stages of use, when the friction material and the interlayer were completely worn, and the friction occurred between the pin and the back plate. The coefficient of friction took on values reaching up to over 0.8 (for samples obtained from premium car brake pads). In an attempt to find the cause of this increase, analysis of the behavior of metal elements in contact was performed. The normal force generates the contact pressure which causes surface stresses [46,47]. These, in turn, can cause deformations, including plastic ones [48]. If the surfaces move in relation to each other, fragments of the material may be “torn” from them—this is called adhesive wear. A similar phenomenon was noticed in many other studies in which steel was combined with steel [49,50,51,52]. In order to check whether there was adhesion in the tested samples, as was also the case previously, SEM was used. The obtained photograph is shown in Figure 12. As can be seen, in many places, there are visible losses, delamination and deformation of the material caused by dry friction. This confirms that the adhesion between the friction pair was responsible for such a high increase in the value of the friction coefficient.

After conducting the tests on the T-11 station, each sample was washed, dried and weighed. Each set of samples cut from the same brake pad was assessed in terms of the mass lost from before the test. Arithmetic average values (z_av_) and standard deviations of the measurements were given by:

(22) $$S_{d}=\sqrt{\frac{\sum_{i=1}^{3}{\left(z-\bar{z}\right)}^{2}}{2}}$$

where: *z*—weight loss of a single sample. These values are compiled in Table 7.

**Figure 13:**
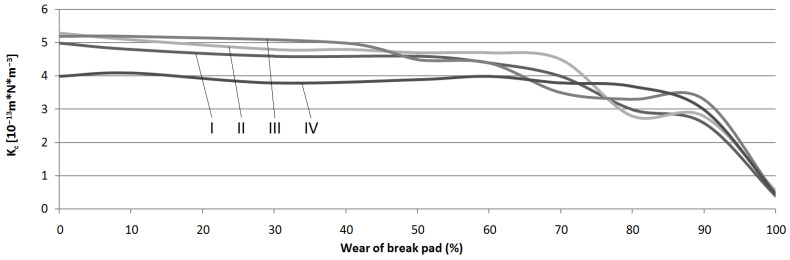
Friction wear rate values in all samples.

The abrasive wear rate was calculated using a modified Archard’s equation [53]:

(23) $$K_{c}=\frac{V}{SN}$$

where: *V*—volume of wear material (m^3^); *S*—sliding distance; *N*—load. Using Archimedes’ law, densities of the friction material were determined. The densities were similar in all brake pad groups and were as follows: friction material—2850 kg/m^3^; interlayer—2248 kg/m^3^; metal back plate—7845 kg/m^3^. These calculations made it possible to measure the density of the wear debris. The obtained results were applied in the equation, producing the *K_c_* values for each sample. The Lagrange rule (described above) was also used to complete the missing data. The final calculations are presented in Figure 13.

The results showed that the intensity of the wear changed along with the degree of wear. The *K_c_* coefficient significantly decreased in all samples, especially in the end-of-life phase. This indicated that the fastest wear was observed in brand-new brake pads and those that were used only for a short or normal time (up to 60–70%). This was due to the fact that the pads were designed in such a way as to provide maximum braking force without the risk of overheating. Therefore, a compromise is needed between the abrasive wear rate and the generated friction force. Currently, manufacturers, due to better vehicle performance, increase COF at the expense of wear rate [54].

The lowest *K_c_* values were observed in those brake pads that were completely worn. Coronado explained that in the case of hard materials, a lot of the kinetic energy of movement is used to remove material in form of microchips [42]. This is responsible for a significant increase in the coefficient of friction (as was observed above) with a simultaneous decrease in the coefficient of abrasive wear. Avient et al. obtained similar results [55]. Moreover, they found that the influence of wear products’ presence between the cooperating surfaces might be the reason for increased friction force. The frictional energy in that case is used to deform and grind the wear products, which also reduces *K_c_* coefficient values.

## 4. Conclusions

This article presents the results of tests concerning the coefficient of friction and abrasive wear rate of the working parts of brake systems. A total of 111 samples collected from brake pads were tested. Four different models of genuine brake pads were used, both brand new and at different degrees of wear, intended for different types of cars. A single-factor analysis of variance method was used to properly analyze the results. The Levene test made it possible to check the degree of homogeneity of the variations in the groups. Lagrange interpolation made it possible to assume approximate values of the missing measurements. The final results made it possible to assess the effects of the time of use of brakes on the coefficient of friction and the abrasive wear rate. It was determined that:

(1) The coefficient of friction for all of the tested brake pads decreased as they were used; the biggest changes in the coefficient of friction depended on the changes in the material composition of the brake pad (depending on the degree of wear, the friction in the system was created by the friction material, the adhesive (interlayer) and the back plate);
(2) The highest values of the coefficient of friction were observed in samples collected from completely worn pads; this was most likely due to the strong adhesion in the friction pair (metal back plate and the cast iron pin);
(3) The brake pads showed the fastest wear when they were brand new, which resulted from the need for run-in between the contact surfaces;
(4) The slowest brake pad wear was observed when the back plate constituted the contact surface.

From the safety point of view, the information about the value of the coefficient of fiction is of particular importance, as it has a direct impact on the stopping force of the vehicle. The value of the *K_c_* coefficient indicates the lifetime of the brake pad. Information on the specificity of changes of both of these parameters are valuable to both producers and car users.

## Figures and Tables

**Table 1:** List of obtained test samples.

Degree of Wear (%)	0	10	20	30	40	50	60	70	80	90	100
Number of samples in group 1	3	3	0	3	0	3	3	3	3	3	3
Number of samples in group 2	3	0	3	3	3	3	3	3	3	3	3
Number of samples in group 3	3	0	0	3	3	3	3	3	3	3	3
Number of samples in group 4	3	0	3	0	3	3	3	3	3	3	3

**Table 2:** Composition of individual samples and their layers.

Brake Pads Group No.	Layer	Composition (% of Total Mass)
I	Friction material	Phenolic resin—29.69%; steel fibers—3.81%; glass fiber—7.99%; cast iron fibers—3.48%; silicon carbide—0.92%; zeolites—5.19%; zinc oxide—1.68%; graphite—2.18%; copper—6.46%; barite—15.63%; silicates—9.46%; magnesium oxides—15.52%
	Binder layer (interlayer)	Phenolic resin—41.99%; steel fibers—2.34%; cast iron fibers—1.58%; glass fiber—3.28%; silicon carbide—0.37%; zeolites—4.34%; zinc oxide—1.51%; graphite—2.18%; barite—14.39%; silicates—5.40%; magnesium oxides—16.15%
	Support plate (backplate)	C—0.17%; Mn—1.41%; Si—0.21%; P—0.04%; S—0.02%; Fe—98.14%
II	Friction material	Phenolic resin—18.14%; steel fibers—3.95%; glass fiber—7.43%; cast iron fibers—2.40%; silicon carbide—0.98%; zeolites—5.68%; zinc oxide—1.85%; graphite—2.86%; copper—6.22%; barite—18.58%; silicates—8.36%; magnesium oxides—17.06%; rubber particles—6.22%
	Binder layer (interlayer)	Phenolic resin—38.33%; steel fibers—2.38%; cast iron fibers—1.45%; glass fiber—3.47%; silicon carbide—0.38%; zeolites—4.40%; zinc oxide—1.27%; copper—6.70%; graphite—2.02%; barite—13.40%; silicates—6.74%; magnesium oxides—17.11%; rubber particles—2.36%
	Support plate (backplate)	C—0.16%; Mn—1.34%; Si—0.18%; P—0.02%; S—0.03%; Fe—98.13%
III	Friction material	Phenolic resin—16.85%; steel fibers—4.17%; glass fiber—7.40%; cast iron fibers—2.64%; silicon carbide—0.82%; zeolites—3.80%; zinc oxide—2.33%; graphite—2.85%; copper—8.23%; barite—18.47%; silicates—8.81%; magnesium oxides—16.94%; rubber particles—6.68%
	Binder layer (interlayer)	Phenolic resin—37.11%; steel fibers—2.53%; cast iron fibers—1.39%; glass fiber—3.84%; silicon carbide—0.48%; zeolites—4.27%; zinc oxide—1.06%; copper—6.48%; graphite—2.18%; barite—14.65%; silicates—6.66%; magnesium oxides—17.28%; rubber particles—2.05%
	Support plate (backplate)	C—0.18%; Mn—1.41%; Si—0.26%; P—0.02%; S—0.02%; Fe—98.11%
	Friction material	Phenolic resin—30.74%; steel fibers—3.3%; glass fiber—6.09%; cast iron fibers—3.64%; silicon carbide—1.5%; zinc oxide—1.41%; graphite—2.84%; copper—6.03%; barite—17.94%; silicates—9.46%; magnesium oxides—17.04%
IV	Binder layer (interlayer)	Phenolic resin—46.39%; steel fibers—2.25%; cast iron fibers—1.42%; glass fiber—2.28%; silicon carbide—0.41%; zinc oxide—1.42%; copper—6.01%; graphite—2.08%; barite—14.25%; silicates—6.50%; magnesium oxides—16.42%
	Support plate (backplate)	C—0.18%; Mn—1.39%; Si—0.22%; P—0.03%; S—0.02%; Fe—98.15%

**Table 3:** Average Rockwell hardness of samples.

Brake Pad Wear (%)	Rockwell Hardness (HRC)			
	I	II	III	IV
10	55	57	57	54
20	54	-	-	-
30	-	54	-	55
40	53		56	-
50	-	52	54	54
60	52	54	55	54
70	53	52	54	52
80	53	51	52	52
90	48	49	47	49
100	62	62	61	64

**Table 4:** Calculated coefficients of friction for particular samples.

Brake Pad Wear (%)	Test No.	Coefficient of Friction of Brake Pad Samples			
		I	II	III	IV
10	1	0.391	0.469	0.441	0.342
	2	0.385	0.459	0.544	0.361
	3	0.396	0.455	0.458	0.381
20	1	0.389	-	-	-
	2	0.446	-	-	-
	3	0.402	-	-	-
30	1	-	0.416	-	0.388
	2	-	0.497	-	0.405
	3	-	0.440	-	0.339
40	1	0.359	0.367	0.438	-
	2	0.386	0.456	0.514	-
	3	0.374	0.462	0.511	-
50	1	-	0.414	0.481	0.359
	2	-	0.406	0.426	0.341
	3	-	0.380	0.506	0.429
60	1	0.355	0.382	0.412	0.376
	2	0.357	0.434	0.428	0.341
	3	0.339	0.417	0.477	0.402
70	1	0.331	0.352	0.414	0.348
	2	0.294	0.377	0.389	0.340
	3	0.299	0.348	0.462	0.416
80	1	0.281	0.343	0.297	0.332
	2	0.277	0.308	0.329	0.368
	3	0.254	0.315	0.292	0.386
90	1	0.134	0.168	0.264	0.315
	2	0.171	0.207	0.286	0.352
	3	0.143	0.246	0.210	0.365
100	1	0.192	0.221	0.218	0.199
	2	0.169	0.323	0.262	0.150
	3	0.129	0.215	0.274	0.194

**Table 5:** Single-factor analysis of variance calculation results.

Sample Group	Source of Variation	*D_f_*	*SS*	*MS*	*F_f_*	*p*
I	qualitative factor	10	0.832516	0.083252	26.513	0
	random error	22	0.006908	0.000314	-	-
	total	32	0.839424	-	-	-
II	qualitative factor	10	0.726189	0.072619	64.853	0
	random error	22	0.024634	0.001120	-	-
	total	32	0.750823	-	-	-
III	qualitative factor	10	0.675614	0.067561	57.696	0
	random error	22	0.025762	0.001171	-	-
	total	32	0.701375	-	-	-
IV	qualitative factor	10	0.552307	0.055231	71.031	0
	random error	22	0.017106	0.000778	-	-
	total	32	0.569413	-	-	-

**Table 6:** Levene test results.

	Sample Group			
	I	II	III	IV
*F_Lev_*	1.814206	2.681519	0.782742	1.766654

**Table 7:** Mass loss according to the results of the conducted friction tests.

Brake Pad Wear (%)	Average Mass Loss of Brake Pad Samples (g):							
	I		II		III		IV	
	z_av_	S_d_	z_av_	S_d_	z_av_	S_d_	z_av_	S_d_
0	0.6989	±0.099	0.7409	±0.159	0.7269	±0.190	0.5591	±0.126
10	0.6710	±0.136	-	-	-	-	-	-
20	-	-	0.7129	±0.161	-	-	0.5731	±0.096
30	0.6430	±0.164	0.6710	±0.107	0.7269	±0.193	-	-
40	-	-	0.6710	±0.101	0.6989	±0.150	0.5312	±0.174
50	0.6430	±0.101	0.6570	±0.184	0.6290	±0.178	0.5451	±0.155
60	0.6150	±0.155	0.6570	±0.095	0.6150	±0.170	0.5591	±0.144
70	0.5591	±0.148	0.6290	±0.153	0.4892	±0.143	0.5312	±0.105
80	0.4193	±0.168	0.3914	±0.188	0.4613	±0.096	0.5172	±0.129
90	0.3634	±0.158	0.3914	±0.159	0.4613	±0.186	0.4193	±0.166
100	0.0559	±0.125	0.0782	±0.097	0.0643	±0.141	0.0629	±0.113

## Data Availability

The data presented in this study are available on request from the corresponding author. At the time the project was carried out, there was no obligation to make the data publicly available.
